# Comparative genomic and functional analyses of *Microbacterium paraoxydans* BHS25 reveal key metabolic adaptations for survival in arsenic-contaminated soil ecosystems

**DOI:** 10.1186/s12864-025-11811-7

**Published:** 2025-07-09

**Authors:** Ayman Bin Abdul Mannan, Momtaz Zamila Bukharid, M. Anwar Hossain, Munawar Sultana

**Affiliations:** Department of Microbiology, University of Dhaka, Dhaka, 1000 Bangladesh

**Keywords:** *Microbacterium*, Comparative genomics, Metabolic diversity, Arsenic transformation, Metal tolerance

## Abstract

**Background:**

*Microbacterium paraoxydans* is known for its potential in bioremediation and biotechnological applications, including promoting plant growth. However, research on this bacterium in Bangladesh has been limited and until now no reported complete genome of *M. paraoxydans* is available from this country. In this study, we have reported the complete genome of *M. paraoxydans* BHS25, the first case in Bangladesh, isolated from arsenic-contaminated soil in Bogura.

**Results:**

Complete genome analysis revealed that BHS25 was closely related to *Microbacterium paraoxydans* LTR1 from Russia, which itself showed similarity to a strain found at the International Space Station, reported to be resistant to extreme conditions. BHS25 possessed a genome of 3.49 Mb with a GC content of 70.12%, comprising 3,415 protein-coding genes, 47 tRNA genes, and 5 rRNA genes. It carried various heavy metal resistance genes and gene islands, such as *ars*C, *ars*B, and *acr*3 for arsenic detoxification/transformation, as well as *czc*D and *cop*B for resistance to cadmium, zinc, cobalt, and copper. The arrangement of the arsenic resistance genes showed similarity to that in other reported *Microbacterium* strains, although pangenome and ANI analyses indicated considerable genetic diversity within the species. Additionally, the presence of *van*Y within the *van*B cluster suggested potential vancomycin resistance. Metabolic pathway analyses revealed that BHS25 was well adapted, with different carbohydrate and amino acid metabolism, secondary metabolite biosynthesis, and xenobiotic degradation capabilities. The unique notable anabolic pathways were *streptomycin biosynthesis* with 14 associated genes, *novobiocin biosynthesis* and *tropane*,* piperidine*, and *pyridine alkaloid biosynthesis* (8 genes each), as well as *monobactam biosynthesis*, *prodigiosin biosynthesis*, and *penicillin* and *cephalosporin biosynthesis*, suggesting a potential for production of antimicrobials. Furthermore, it showed an auxin biosynthesis pathway for plant growth, further demonstrating its biotechnological potential.

**Conclusion:**

This research identified *Microbacterium paraoxydans* BHS25 as a promising candidate for bioremediation and sustainable environmental management, offering insights into microbial adaptation to challenging environments and potential solutions for pollution encounters.

**Supplementary Information:**

The online version contains supplementary material available at 10.1186/s12864-025-11811-7.

## Introduction

Environmental contamination remains a critical global challenge, with profound implications for both ecosystems and human health. Among the various pollutants that threaten our planet, arsenic is particularly concerning because of its widespread presence in water and soil, especially in regions such as Bangladesh. Arsenic, a toxic metalloid, poses severe risks to agriculture and human health, with exposure linked to skin lesions, neurological disorders, and cardiovascular diseases [1]. Furthermore, arsenic contamination disrupts the ecological balance, negatively impacting biodiversity and ecosystem functionality [2].

In addition to arsenic, other heavy metals, such as lead, mercury, cadmium, and chromium, also contribute significantly to environmental pollution. Lead contamination, which often originates from industrial processes, batteries, and paints, is highly toxic, especially to children, and can lead to cognitive impairments, kidney damage, and developmental delays [3]. Cadmium, which is commonly found in fertilizers, industrial effluents, and mining waste, is a known carcinogen that affects kidney function and bone health [3, 4]. Chromium, particularly its hexavalent form, is released from leather tanning and electroplating industries and is toxic to aquatic organisms, soil microbes, and humans, causing respiratory issues, skin damage, and cancer [5]. To address these issues, innovative and sustainable bioremediation strategies are urgently needed to mitigate the detrimental impacts of arsenic and other heavy metal pollutants.

Microorganisms, which are often overlooked, play a central role in bioremediation because of their remarkable adaptability to harsh environments. Many bacteria have evolved sophisticated mechanisms to resist and even transform toxic compounds, enabling them to thrive in contaminated settings. Of particular interest are bacteria capable of degrading xenobiotics—complex organic pollutants foreign to the biological system—and producing secondary metabolites, such as antibiotics, which inhibit the growth of competing microorganisms. These capabilities position certain bacteria as key players in natural bioremediation processes. One such bacterium, *Microbacterium paraoxydans* BHS25, has been isolated from arsenic-contaminated soil in Bangladesh, a region associated with significant arsenic pollution [6]. Members of the *Microbacterium* genus are known for their ability to survive in diverse and often hostile environments, including those polluted with heavy metals. The bacterial genus *Microbacterium* is of particular interest because of its multifunctionality, which includes heavy metal resistance, antibiotic production, xenobiotic degradation, and auxin production—a plant growth-promoting hormone [7, 8]. Understanding the genetic basis of these functions could open new avenues for bioremediation and sustainable agricultural practices.

In Bangladesh, *Microbacterium paraoxydans* is likely underreported due to a lack of focused research on environmental microbiology and limited resources for genomic studies. Microbiological research often prioritizes more well-known pathogens or agricultural organisms with direct impacts on public health or industry. Additionally, the presence of *M. paraoxydans* in specific, less-studied environmental niches, such as contaminated soils or water, may not be a primary research focus. The limited access to advanced sequencing technologies and the focus on more pressing public health concerns could explain the small number of whole genomes reported from Bangladesh. However, as interest in environmental microbiomes and pollution-related studies grows, *M. paraoxydans* may attract more attention in the future.

Whole-genome sequencing (WGS) generates a comprehensive map of an organism’s DNA, enabling the identification of genes responsible for traits such as heavy metal tolerance, antibiotic resistance, virulence, and disease causation. By comparing sequences against specialized databases (e.g., CARD for antibiotic resistance genes and VFDB for virulence factors), researchers can rapidly assess an isolate’s resistance mechanisms and pathogenic risks.

Comparative genomics extends this by analyzing multiple strains simultaneously. This approach highlights genomic diversity, uncovers unique traits (e.g., novel metabolic functions or resistance genes), and predicts evolutionary or environmental relationships. Pangenome analysis categorizes genes into the core genome (shared by all strains) and the accessory genome (strain-specific), clarifying phylogenetic relationships. Synteny analysis examines the order and arrangement of gene clusters and operons, revealing genomic adaptability.

Metabolic analysis predicts the substrates a bacterium can metabolize and the compounds it can produce. Bioinformatics tools such as RAST, PATRIC, KEGG, and EggNOG-mapper identify metabolic pathways and subsystems by linking genes to their functional roles. This analysis aids in evaluating the biotechnological applications of bacterial isolates, such as their potential for bioremediation or industrial processes.

## Objective of the study

This study aims to leverage functional and comparative genomics and bioinformatics analyses to explore the multifunctionality of *Microbacterium paraoxydans* BHS25 isolated from arsenic-contaminated soil in Bangladesh. By identifying and characterizing genes involved in heavy metal resistance, antibiotic production, xenobiotic degradation, and plant growth promotion, this research seeks to provide new insights into the potential biotechnological applications of this bacterium in environmental remediation and sustainable agriculture here locally in Bangladesh.

## Materials and methods

### Sample collection, isolation, and parameter measurement

Several tubewells in the Bogura district of Bangladesh, (24°50′53.0808″N, 89°22′22.6668″E) were identified by the Department of Public Health Engineering (DPHE) as being contaminated with arsenic concentrations ≥ 50 µg/L—exceeding the World Health Organization (WHO) permissible limit. Surface soil samples (0–15 cm) from the surrounding area of such a contaminated tubewell from Gabtali Upazila in Bogura were collected for microbiological investigation.

Soil pH was measured using the electrometric method with a pH meter equipped with a combination glass electrode. The total arsenic content and soil cations were determined using a flame atomic absorption spectrophotometer [9] with a hydride generation system. Anion concentrations (NO_4_^–^ and SO_4_^2–^) were analyzed in untreated soil samples using ion chromatography.

To isolate arsenic-resistant bacteria, the soil samples were enriched in a heterotrophic enrichment medium supplemented with 2 mM sodium arsenite (NaAsO_2_) and incubated for four weeks [7, 10, 11]. Following enrichment, serial dilutions were plated on heterotrophic agar containing sodium arsenite, and the plates were incubated at 25 °C for 24–48 h. Distinct colonies were selected and purified for further analysis.

### Confirmation of arsenic resistance via MIC

The isolates were grown in 5 mL of heterotrophic broth at 30 °C and 120 rpm until the optical density at 600 nm reached 0.1. Then, 70 µL of this broth, with various doses of arsenite, As (III) as NaAsO2 (0 to 32 mM), was added to each well of a 96-well plate. A 5 µL bacterial inoculum (OD600 = 0.1) was added to each well, followed by autoclaved deionized water to bring the final volume to 100 µL. For the negative control, a row was set up with only As (III) medium, without bacteria. The plate was incubated at 30 °C for 24 h. Afterwards, the cell density and bacterial growth were measured using a spectrophotometer at 600 nm. From the isolates, BH25 was selected for molecular investigation due to its well-defined growth and tolerance to supplemented arsenite.

### DNA extraction, whole-genome sequencing, quality control, assembly and identification

The bacterial DNA was extracted from the pure culture of BHS25 via Promega Wizard^®^ Genomic DNA Purification Kit according to its manual. The whole-genome sequencing of BHS25 was conducted via the Illumina platform on the Illumina MiniSeq system, generating 150 bp paired-end FASTQ files. Quality assessment of the raw reads was carried out via FastQC (v0.11.9) [12]. Adapter sequences and low-quality bases were trimmed via Trimmomatic (v0.39) [13], with settings applied for a minimum average quality score of 20 and a minimum read length of 50 bp. After trimming, the high-quality reads were assembled de novo via SPAdes v3.15.4 [14].

For isolate identification, the assembled genomes were analyzed via BLASTP, the KmerFinder tool from the Center for Genomic Epidemiology, which employs a k-mer algorithm for species identification [15]. Additionally, identification and validation of the isolates were performed via average nucleotide identity (ANI) calculations. ANI values were determined via the ANI Calculator tool [16], which was developed by the Environmental Microbial Genomics Laboratory at the Georgia Institute of Technology. The ANI comparison of the *Microbacterium paraoxydans* isolates was conducted against reference genomes of *Microbacterium* species available in public databases.

### Genome annotation and genomic organization mapping

Given the limited availability of whole-genome sequences of *Microbacterium paraoxydans* in public databases, multiple annotation pipelines were employed to ensure comprehensive coverage of all coding sequences (CDSs) and genes. The assembled genomes of the isolates were annotated via various tools, including Prokka [17], RAST [18] and the Patric server [19].

### Comparative genomic analyses

To explore the evolutionary relationships and genetic diversity of *Microbacterium paraoxydans* isolates, several comparative genomic and phylogenetic analyses were performed. Codon tree analysis was conducted via the PATRIC Codon Tree service [20] to infer phylogenetic relationships among the isolates and other closely related species. This method involves constructing a maximum likelihood tree based on the alignment of 100 single-copy genes, providing insights into the evolutionary lineage of the isolates.

Average nucleotide identity (ANI) analysis was performed via the fastANI [21] tool to measure the genomic similarity between the isolates and related species. The resulting ANI heatmap visually represented the genomic distances, allowing for a comparative assessment of the isolates within the context of known *Microbacterium* species.

Pangenome analysis was conducted to assess the genomic diversity and identify core and accessory genes among the isolates. This analysis classified genes into core, accessory, and unique categories, shedding light on the genetic variability and potential functional adaptations of the isolates. These combined analyses provided a comprehensive understanding of the evolutionary relationships, genetic diversity, and functional potential of *Microbacterium paraoxydans* isolates, highlighting their adaptability to arsenic-contaminated environments and their potential for biotechnological applications [22].

### Analysis of heavy metal resistance, antimicrobial resistance, virulence factors and metabolic pathways

The antimicrobial resistance genes were investigated via the Comprehensive Antibiotic Resistance Database [23] (CARD) and ResFinder [24]. To explore virulence factors, the VFDB database [25] was utilized, providing a manually curated resource for identifying virulence factors in pathogens affecting humans and animals. Metabolic functions were predicted through pathway analysis via the KEGG database [26] and COG (Clusters of Orthologous Groups) database [27], whereas metal resistance genes were analyzed via BacMet [28], a bioinformatics resource for antibacterial biocide- and metal resistance genes.

### Synteny analysis

Synteny analysis was performed to investigate the genomic organization and conservation of key genes related to arsenic resistance within different *Microbacterium paraoxydans* isolates. This analysis involved comparing the gene order and arrangement in the genomes to identify conserved synteny blocks and potential genomic rearrangements associated with these functional genes via the comparative system of the Patric server [29].

## Results

### Geochemical properties of soil sample

The soil sample, which was collected from Gabtali Upazila in Bogura, Bangladesh (GPS: 24.8824° N, 89.4482° E), had a slightly acidic pH of 6.47. It contained moderate levels of arsenic (0.49 mg/Kg) and iron (462.9 mg/Kg), along with low concentrations of chromium (0.02 mg/Kg), nitrate (2.31 mg/Kg), sulfate (1.891 mg/Kg), and chloride (8.78 mg/Kg). These values reflected a soil environment with trace elements within typical agricultural ranges.

### General features, MIC determination and 16 S rRNA gene sequencing of BHS25 strain

The BHS25 isolate was a Gram-positive, rod-shaped bacterium that formed small, round, colourless colonies on heterotrophic agar plates containing arsenite. It exhibited a minimum inhibitory concentration (MIC) of 15 mM arsenite, significantly higher than levels typically found in nature. Preliminary identification based on 16 S rRNA gene sequencing revealed that BHS25 belonged to the genus *Microbacterium*. Due to the limited studies on arsenic-resistant *Microbacterium* strains in Bangladesh, further characterization was pursued through whole-genome sequencing of BHS25.

### Raw data filtering, draft genome preparation and quality check of the draft genome

The genome assembly of isolate BHS25 resulted in 24 contigs with a total genome length of 3,486,999 base pairs (bp). The GC content of the genome was calculated as 70.12%. The L50 value, an indicator of assembly quality, was found to be 2, indicating that the two largest contigs together accounted for at least half of the total genome length. Furthermore, the N50 value of 788,398 bp highlighted the robustness of the assembly, indicating that the median contig length was close to this value. CheckM analysis returned a coarse consistency of 97.9%, fine consistency of 97.1%, 99.9% completeness, and 0.1% contamination, corresponding to an overall “Good” genome quality.

### Genome identification, annotation and genome organization mapping

**Fig. 1:**
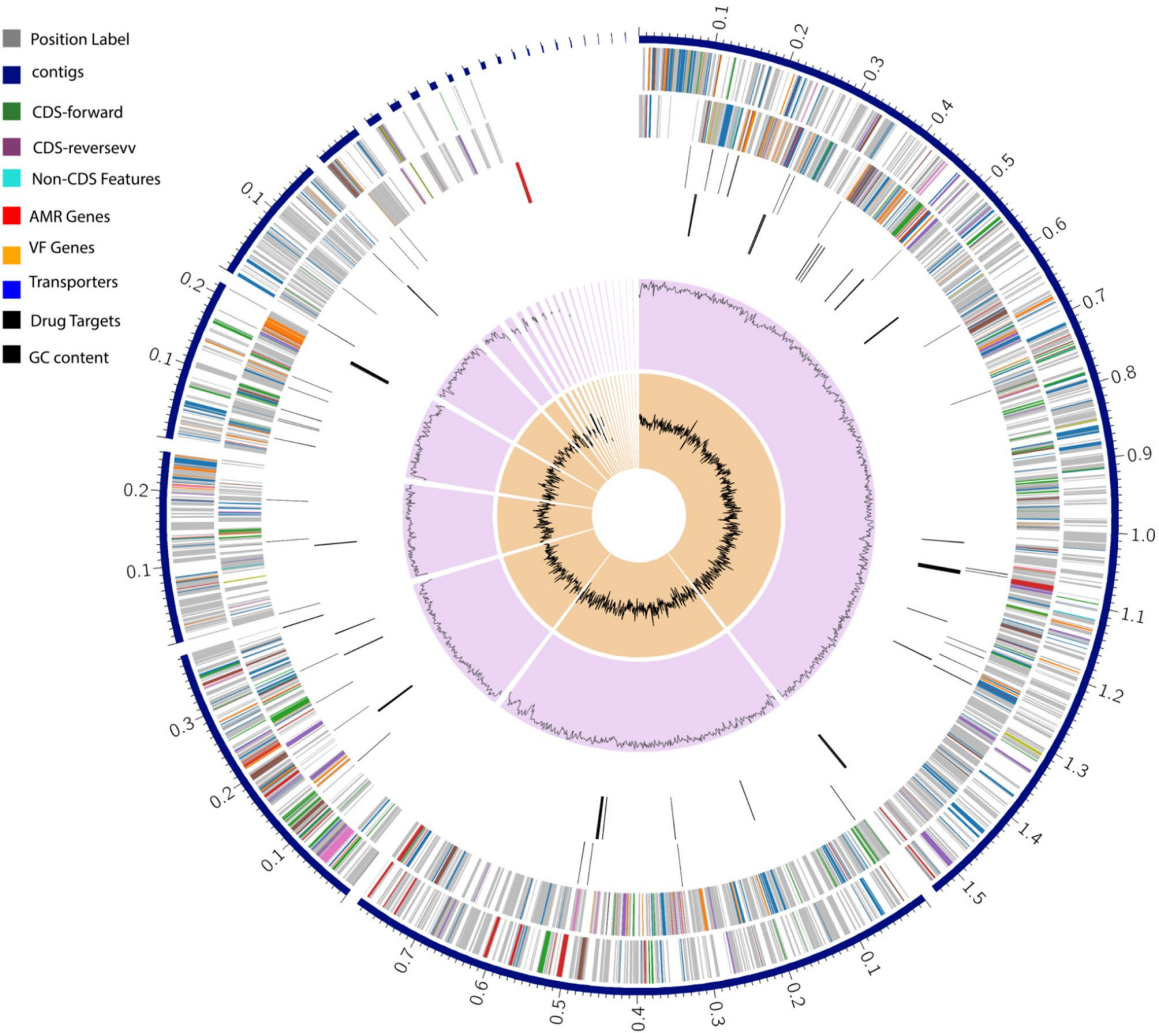
Genome organization mapping of *M. paraoxydans* BHS25, integrating structural, functional, and phylogenetic genomic data. The outermost track depicts the contigs in blue, whereas subsequent tracks include coding sequences (CDSs) on the forward strand (green) and reverse strand (cyan), as well as noncoding features (gray). Specific annotations highlight antimicrobial resistance (AMR) genes (red), virulence factor (VF) genes (orange), transporters (purple), and potential drug targets (blue). The innermost tracks illustrate the GC content distribution (black line) and the phylogenetic relationships of the genomic segments (center)

Genome identification was carried out via BLASTp, KmerFinder, and ANI tools, revealing a high similarity of BHS25 to species within the *Microbacterium* genus. BLASTp analysis revealed that the genome had the highest similarity to that of *Microbacterium paraoxydans* DH1b (accession AYME01000002), with 93% identity and a score of 48,880, followed by *Microbacterium barkeri* 2011-R4 (accession AKVP01000064), with 94% identity and a score of 14,259. KmerFinder further supported these findings, identifying matches to *Microbacterium* sp. *str. ‘China’* (accession NZ_CP027434.1) and *Microbacterium* sp. Y-01 (accession NZ_CP024170.1), with scores of 22,617 and 2,482, respectively. FastANI analysis confirmed a high degree of genome similarity to *M. paraoxydans_*F68SP3C and *M. paraoxydans_*F68SP3A, with identity values of 96.1153% and 96.111%, respectively. Collectively, these results strongly indicated that the genome belongs to the *Microbacterium paraoxydans* species. The genome annotation was performed via Prokka, BV-BRC, and the RAST server. The annotated genome comprised 3,415 protein-coding sequences (CDSs), 47 transfer RNA (tRNA) genes, and 5 ribosomal RNA (rRNA) genes. The organization of the map was represented in a Circos plot in Fig. 1.

### Comparison to other *Microbacterium* isolates (codontree, anitree and pangenomic tree)

**Fig. 2:**
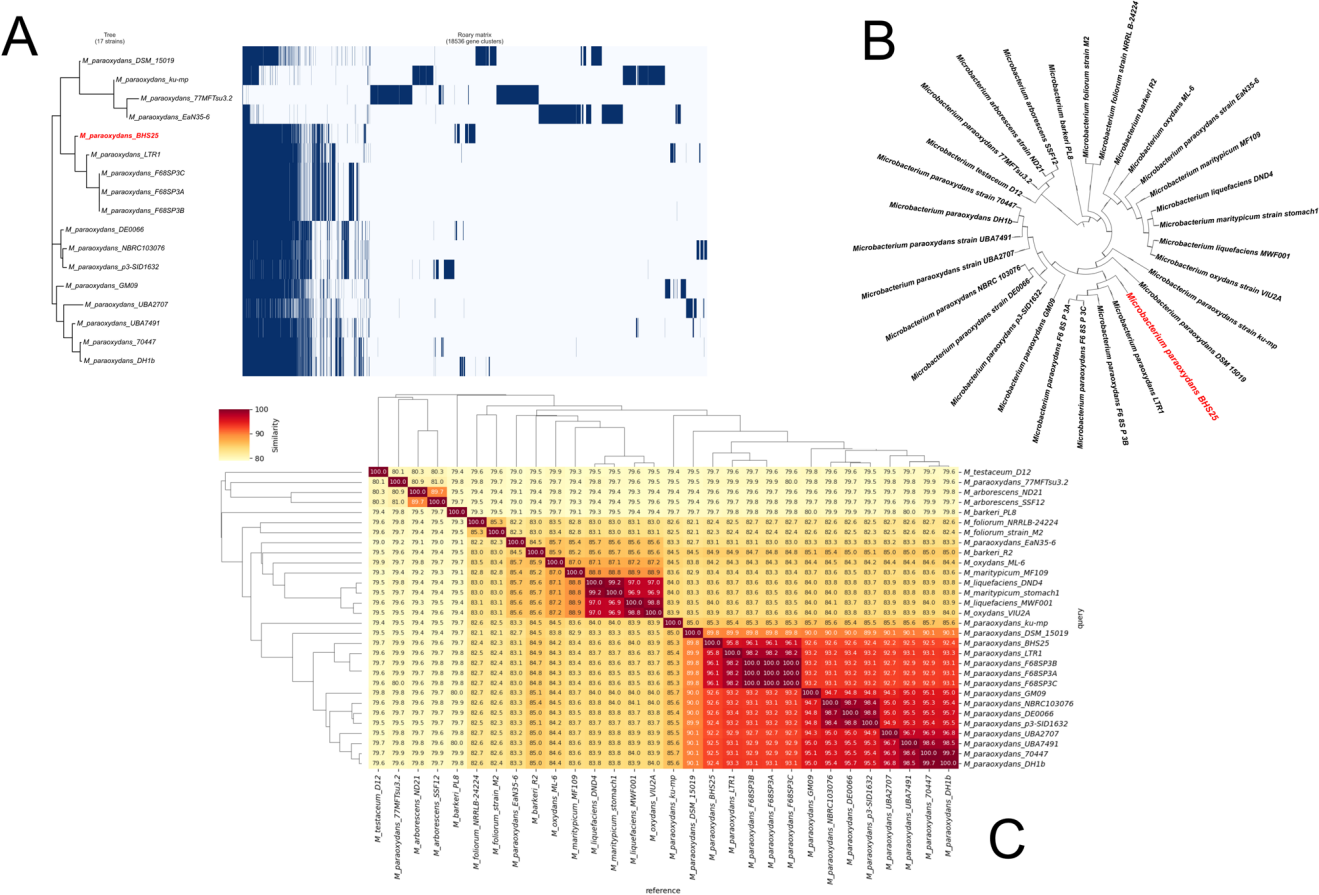
**a** Codontree with the 30 *Microbacterium* isolates **b** pangenomic tree with 17 *Microbacterium* isolates **c** ANI tree with 30 *Microbacterium* isolates

The genome of *Microbacterium paraoxydans* BHS25 was compared with other reported genomes of *M. paraoxydans* and related species from various geographic locations to investigate evolutionary relationships and genetic diversity (Fig. 2a, b, c). Seventeen *M. paraoxydans* genomes reported globally up to 2023 were selected alongside other *Microbacterium* species for phylogenetic tree construction and average nucleotide identity (ANI) analysis. The phylogenetic relationships provided insights into the evolutionary proximity of *M. paraoxydans* BHS25 to other isolates. Furthermore, pangenome analysis was performed on the 17 *M. paraoxydans* genomes to assess genetic similarities and variations based on the presence and absence of genes. This comprehensive genomic comparison highlighted the evolutionary dynamics and functional diversity of *M. paraoxydans*.

*M. paraoxydans* BHS25 (highlighted in red in Fig. 2) presented the closest genetic relationship to *M. paraoxydans* LTR1, a Russian isolate reported in 2021. Additionally, *M. paraoxydans* BHS25 and *M. paraoxydans* LTR1 clustered together with three *M. paraoxydans* F6_8S isolates from the USA collected in 2018 (Fig. 2a, b). This tight clustering suggested strong genetic similarity among these isolates, indicating a shared evolutionary origin. In contrast, most other *Microbacterium* species, except for two outlier *M. paraoxydans* isolates (*M. paraoxydans* EaN35-6 and *M. paraoxydans* 77MFTsu3.2), were located in distinct clades, reflecting their evolutionary divergence from the core *M. paraoxydans* lineage. Notably, *M. paraoxydans* DH1b, *M. paraoxydans* 70,447, *M. paraoxydans* UBA7491, and *M. paraoxydans* UBA2707, highlighting their genetic distance from the primary *M. paraoxydans* clade.

The complementary ANI analysis corroborated these phylogenetic observations. The highest ANI value, approximately 96%, was observed between *M. paraoxydans* BHS25 and *M. paraoxydans* LTR1. Similar high ANI values were recorded between *M. paraoxydans* BHS25 and the three *M. paraoxydans* F6_8S isolates, reinforcing the close genetic relationship among these strains. In contrast, compared with *M. paraoxydans* BHS25, *M. paraoxydans* DH1b and *M. paraoxydans* 70,447, presented ANI values of 92.4%.

Pangenomic analysis further supported these findings, revealing that the four aforementioned isolates (*M. paraoxydans* BHS25, *M. paraoxydans* LTR1, and the three *M. paraoxydans* F6_8S isolates) formed a closely related cluster. In contrast, *M. paraoxydans* DH1b, *M. paraoxydans* 70,447, *M. paraoxydans* UBA7491, and *M. paraoxydans* UBA2707 formed a distinct clade characterized by a divergent gene presence/absence matrix compared with the other isolates. This pangenomic analysis highlighted the genetic diversity within the *M. paraoxydans* species and reinforced the phylogenetic and ANI-based relationships observed.

The pangenome also revealed that the bacteria has 17 core genomes. The core genome of M. paraoxydans BHS25 includes essential genes for transcription (*hrdB*), translation (*rpsO*,* rpmH*,* rpmB*), protein quality control *(clpP1_2)*, and chromosome partitioning *(parA*). It also contains *ettA* (translational regulation), *miaB* (tRNA modification), and multiple tRNA genes (e.g., tRNA-Trp, tRNA-Lys, tRNA-Ala, tRNA-Val, tRNA-Pro), supporting robust protein synthesis.

In contrast, a large number of genes associated with metabolism and resistance were identified in the shell (4,630 genes) and cloud (18,536 genes) genome fractions, highlighting the strain’s genetic diversity and adaptability.

### Heavy metal and antibiotic resistance gene analysis

**Fig. 3:**
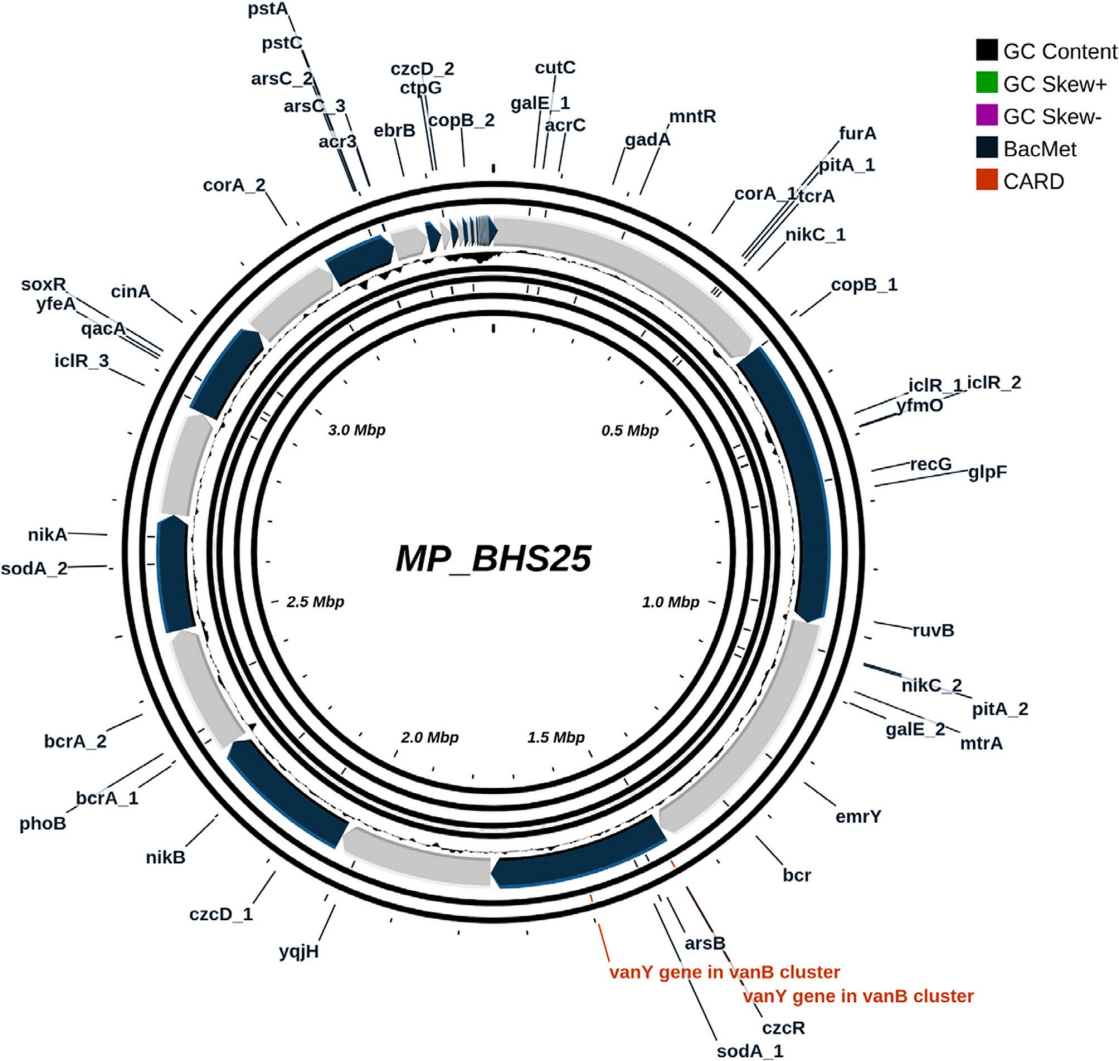
Genome mapping of heavy metal and antibiotic resistance genes on *M. paraoxydans* BHS25

The genome analysis of BHS25 revealed 56 genes linked to heavy metal resistance (Fig. 3; Table 1). Among these genes, notable genes associated with arsenic resistance included *arsC_1*, *arsC_2*, *arsC_3*, *arsB*, and *acr3*. Resistance to cadmium, zinc, and cobalt was associated with the presence of *czcD_1*, *czcD_2*, and *czcR*, whereas copper resistance was attributed to *copB_1*, *csoR*, *cutC*, and *ctpG*. Genes such as *furA*, *mntR*, *sodA_1*, and *sodA_2* could be related to iron and manganese regulation. Nickel resistance was conferred by *nikA*, *nikB*, *nikC_1*, and *nikC_2*, whereas *phoB*, *pstA*, *pstC*, *pitA_1*, and *pitA_2* were attributed to phosphate regulation. The only antibiotic resistance gene identified was against vancomycin, which was conferred by the *vanY* gene located in the *vanB* cluster.

**Table 1:** Antibiotic and heavy metal resistance genes of *M. paraoxydans* BHS25

Resistance Type	Genes
Arsenic Resistance	*arsC_1*,* arsC_2*,* arsC_3*,* arsB*,* acr3*
Cadmium, Zinc, and Cobalt Resistance	*czcD_1*,* czcD_2*,* czcR*
Copper Resistance	*copB_1*,* csoR*,* cutC*,* ctpG*
Iron and Manganese Regulation	*furA*,* mntR*,* sodA_1*,* sodA_2*
Nickel Resistance	*nikA*,* nikB*,* nikC_1*,* nikC_2*
Phosphate Regulation	*phoB*,* pstA*,* pstC*,* pitA_1*,* pitA_2*
Vancomycin resistance	*vanY* gene in *vanB* cluster

### Metabolism insights

**Fig. 4:**
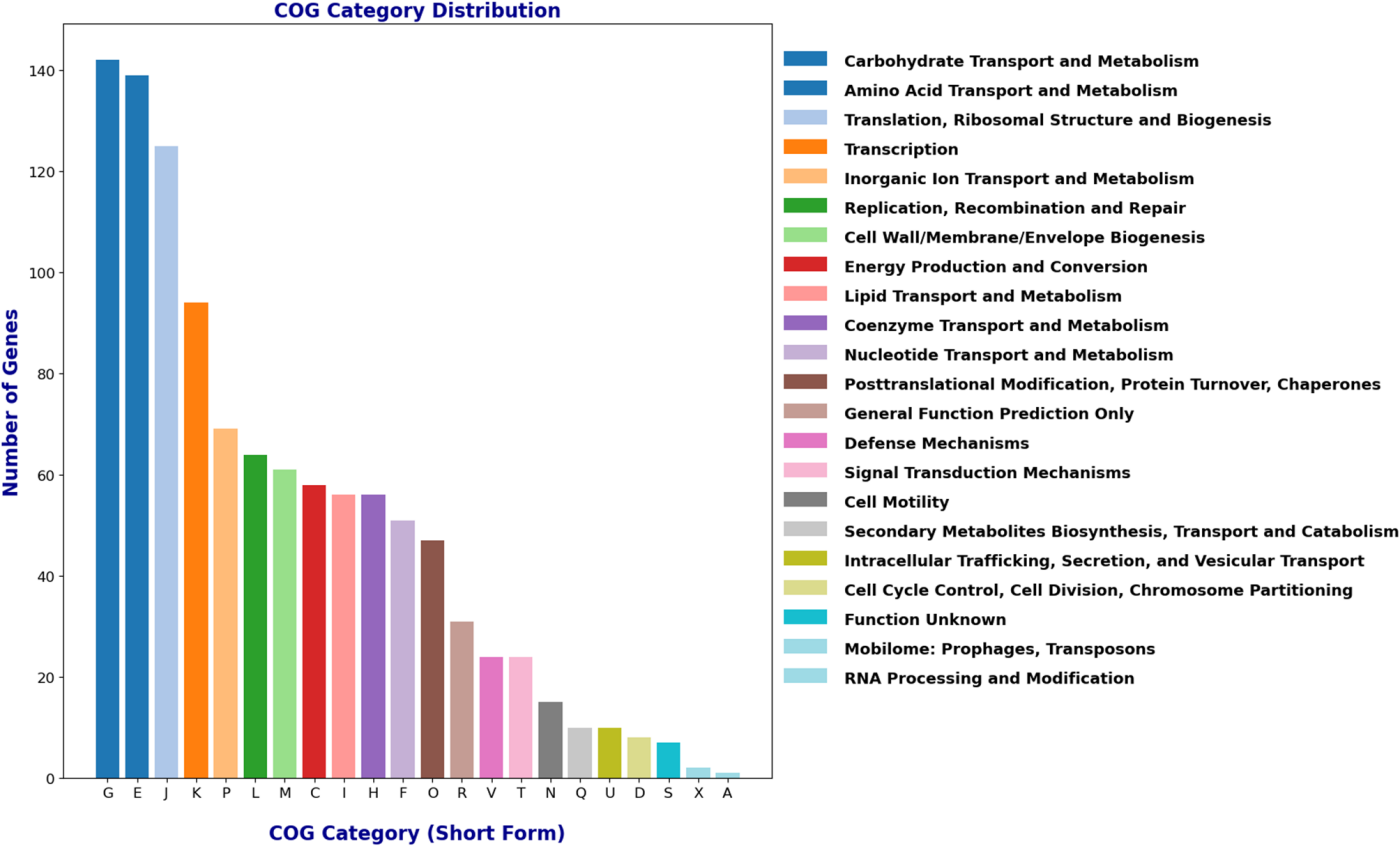
COG (Clusters of Orthologous Groups) category distribution of *M. paraoxydans* BHS25

The metabolism analysis based on the Clusters of Orthologous Groups (COG) category distribution highlighted the organism’s functional diversity and adaptability (Fig. 4). The majority of genes were associated with carbohydrate transport and metabolism, emphasizing their capacity for energy production and utilization. Amino acid transport and metabolism genes were also abundant, reflecting their potential for protein synthesis and nitrogen cycling. A substantial number of genes were involved in translation, ribosomal structure, and biogenesis, indicating a strong ability for protein production. Genes linked to inorganic ion transport and metabolism, as well as energy production and conversion, highlight the ability of organisms to maintain ion homeostasis and energy generation. Moderate representation was observed in categories such as lipid transport, cell wall/membrane biogenesis, and signal transduction mechanisms, indicating structural and regulatory functions. Categories with fewer genes, such as RNA processing, posttranslational modification, and intracellular trafficking, suggested more specialized or secondary roles. This distribution illustrates an organism’s metabolic flexibility, with a focus on essential energy, nutrients, and cellular processes.

**Fig. 5:**
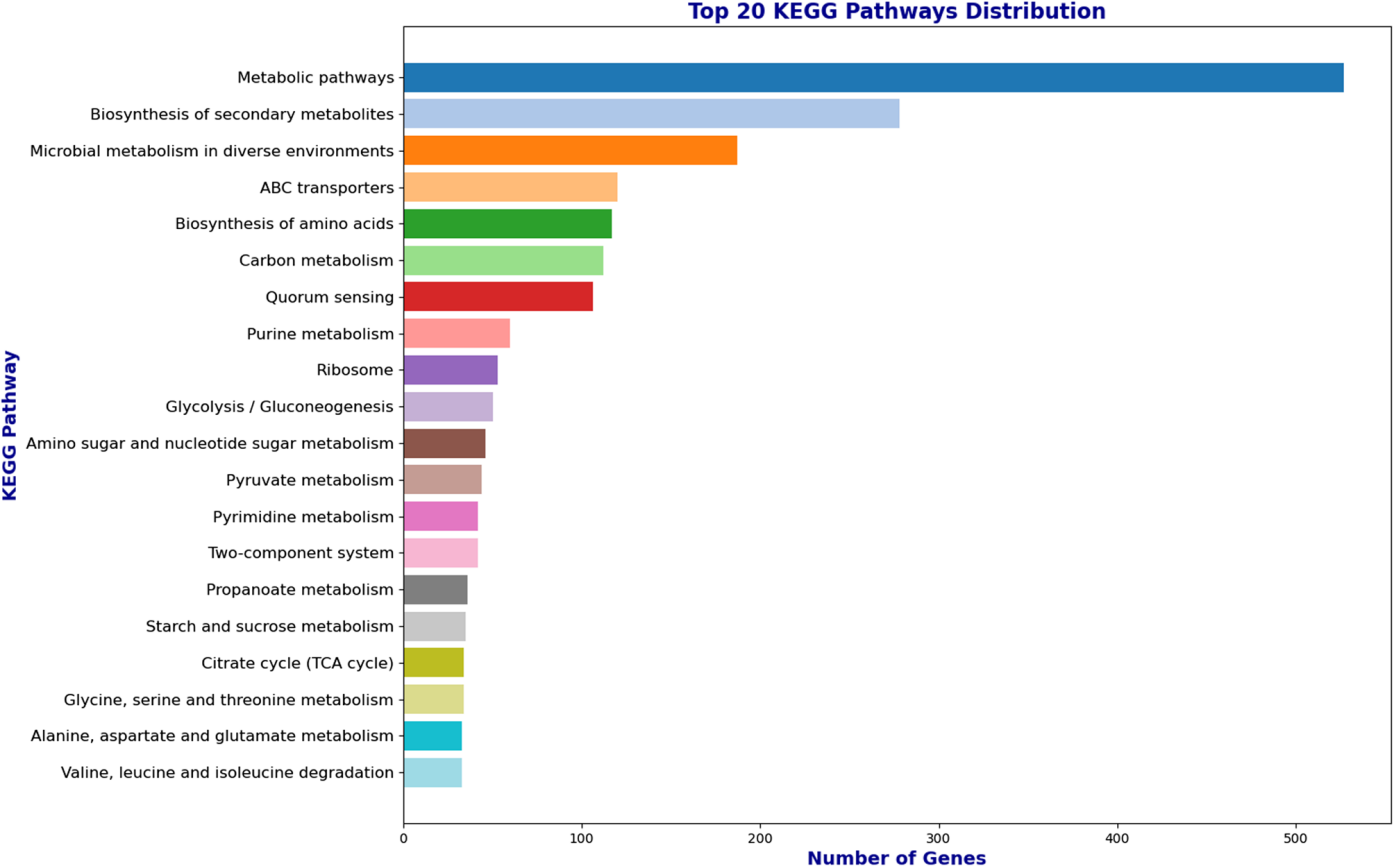
Pathway distribution of *M. paraoxydans* BHS25 retrieved from the KEGG pathway database

The KEGG pathway analysis revealed the organism’s extensive metabolic versatility, with the highest representation in general metabolic pathways, indicating a robust capability for primary metabolism (Fig. 5). The biosynthesis of secondary metabolites was also highly represented, suggesting the organism’s potential for producing complex compounds for adaptation or defence. Pathways associated with microbial metabolism in diverse environments and ABC transporters highlighted its adaptability and efficient nutrient transport systems. Other prominent pathways included amino acid biosynthesis, carbon metabolism, quorum sensing, and purine metabolism, reflecting its ability to synthesize essential building blocks, manage energy flow, and facilitate cell communication. Its moderate representation in glycolysis/gluconeogenesis, ribosome, and pyruvate metabolism underscored its proficiency in energy production and protein synthesis. Additionally, the presence of pathways such as the citrate cycle (TCA cycle), starch and sucrose metabolism, and specific amino acid metabolism pathways illustrated its efficiency in utilizing carbon and nitrogen sources. Overall, the organism demonstrated metabolic diversity and adaptability through the broad distribution of genes across these pathways.

**Fig. 6:**
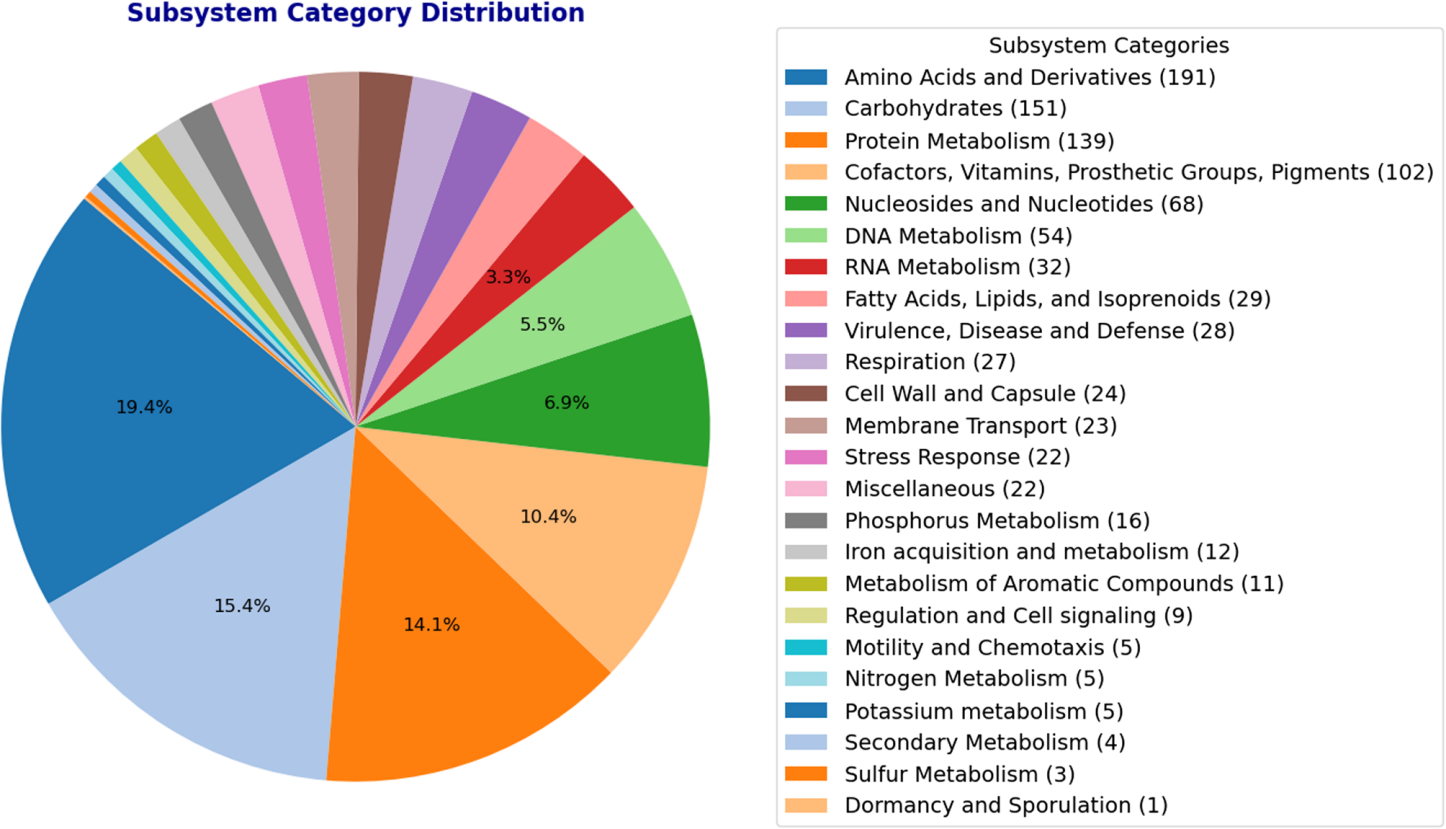
Subsystem Category distributions retrieved from the RAST server

The subsystem category distribution highlighted the metabolic and functional diversity of the organisms (Fig. 6). The largest proportion of genes were associated with “Amino Acids and Derivatives” (19.4%), emphasizing its role in protein synthesis and amino acid metabolism, followed by “Carbohydrates” (15.4%) and “Protein Metabolism” (14.1%), which underscored its capacity for energy production and protein turnover. The significant representation of “Cofactors, Vitamins, Prosthetic Groups, Pigments” (10.4%) and “Nucleosides and Nucleotides” (6.9%) reflected their ability to synthesize essential molecules for metabolic and genetic processes. Categories such as “DNA metabolism” (5.5%) and “RNA metabolism” (3.3%) highlighted its focus on maintaining and expressing genetic information. Smaller but important contributions in “Fatty Acids, Lipids, and Isoprenoids,” “Virulence, Disease, and Defense,” and “Respiration” indicated its adaptability, pathogenic potential, and respiratory activity. Additional pathways involved in nutrient acquisition, such as nitrogen, phosphorus, and iron metabolism, further highlight its metabolic flexibility and environmental adaptability.

### Secondary metabolite biosynthesis and xenobiotic degradation prediction

**Fig. 7:**
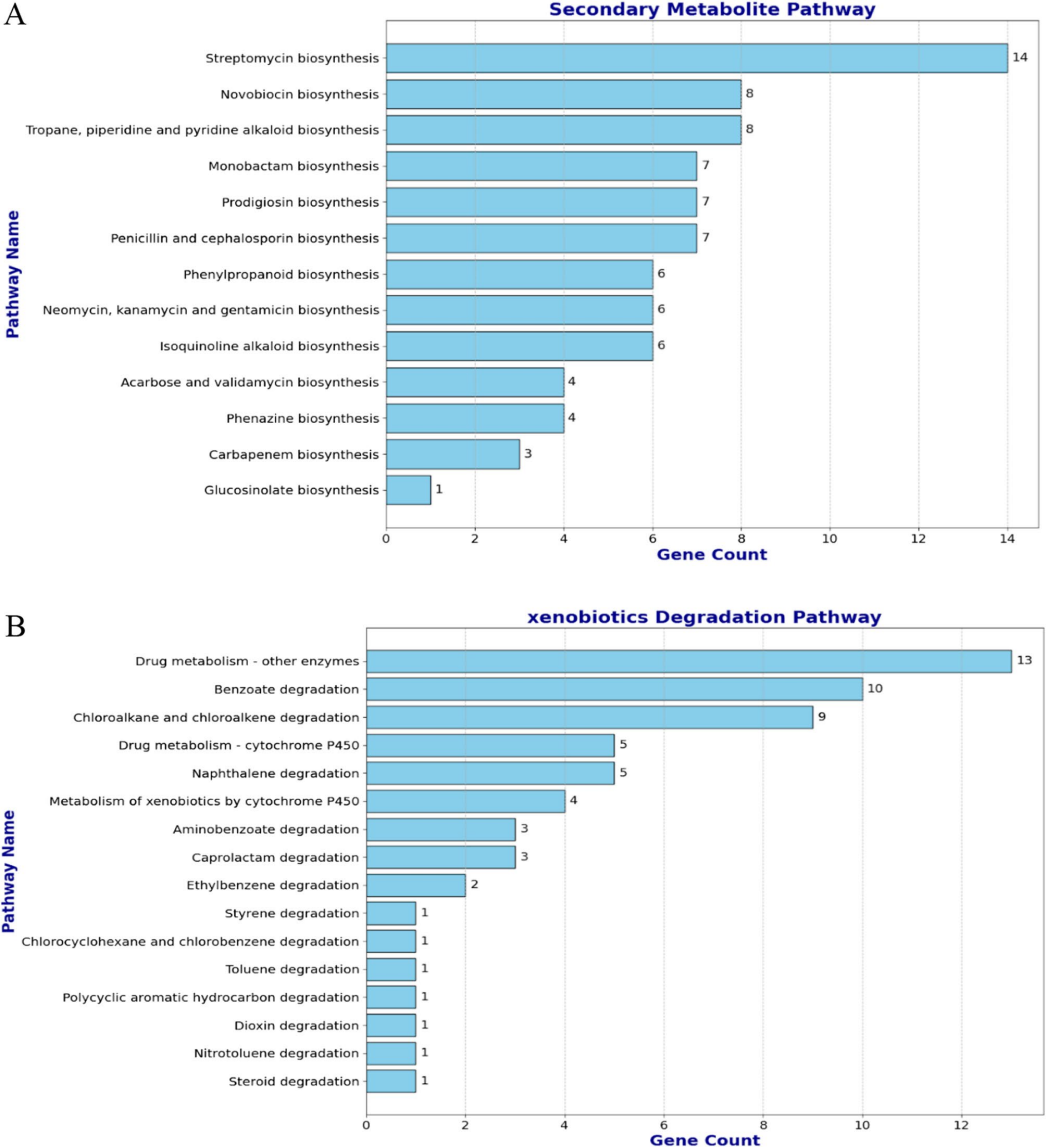
Number of genes related to the corresponding secondary metabolite pathway (**A**) and xenobiotic degradation pathway (**B**)

The analysis of secondary metabolite biosynthesis pathways highlighted the potential of this organism to produce a variety of bioactive compounds (Fig. 7A). The strain harboured genes associated with the production of aminoglycoside antibiotics, including streptomycin (14 genes) and neomycin/kanamycin/gentamicin (6 genes), as well as β-lactam antibiotics such as penicillin/cephalosporin (7 genes) and monobactam (7 genes). It also encoded pathways for alkaloid biosynthesis, including tropane, piperidine, and isoquinoline alkaloids (6–8 genes), and bioactive pigments such as prodigiosin (7 genes) and phenazines (4 genes). Thus, while these findings underscore the organism’s biosynthetic potential, further experimental validation is essential to confirm the significance and activity of these pathways.

BHS25 encoded numerous catabolic pathways for aromatic and halogenated compounds, which are among the most prevalent environmental pollutants (Fig. 7B). For example, 10 gene products map to benzoate degradation namely *oxyS*,* FadA*,* fadJ*,* caiA*,* yhfS*,* echA8*,* paaJ* (with additional genes for aminobenzoate and naphthalene catabolism) and 9 genes to chloroalkane/chloroalkene breakdown *(hcaB*,* aldA*), indicating a robust xenobiotic-degrading potential. The strain also harboured multiple drug/xenobiotic‐metabolism enzymes (including 4–5 cytochrome P450 and some other oxidoreductases), consistent with broad detoxification capability. Overall, most pathways were involved in aromatic and halogenated hydrocarbon catabolism and xenobiotic/drug metabolism, underscoring the bacterium’s metabolic versatility and adaptation to contaminated environments.

### Proteomic comparison and synteny

**Fig. 8:**
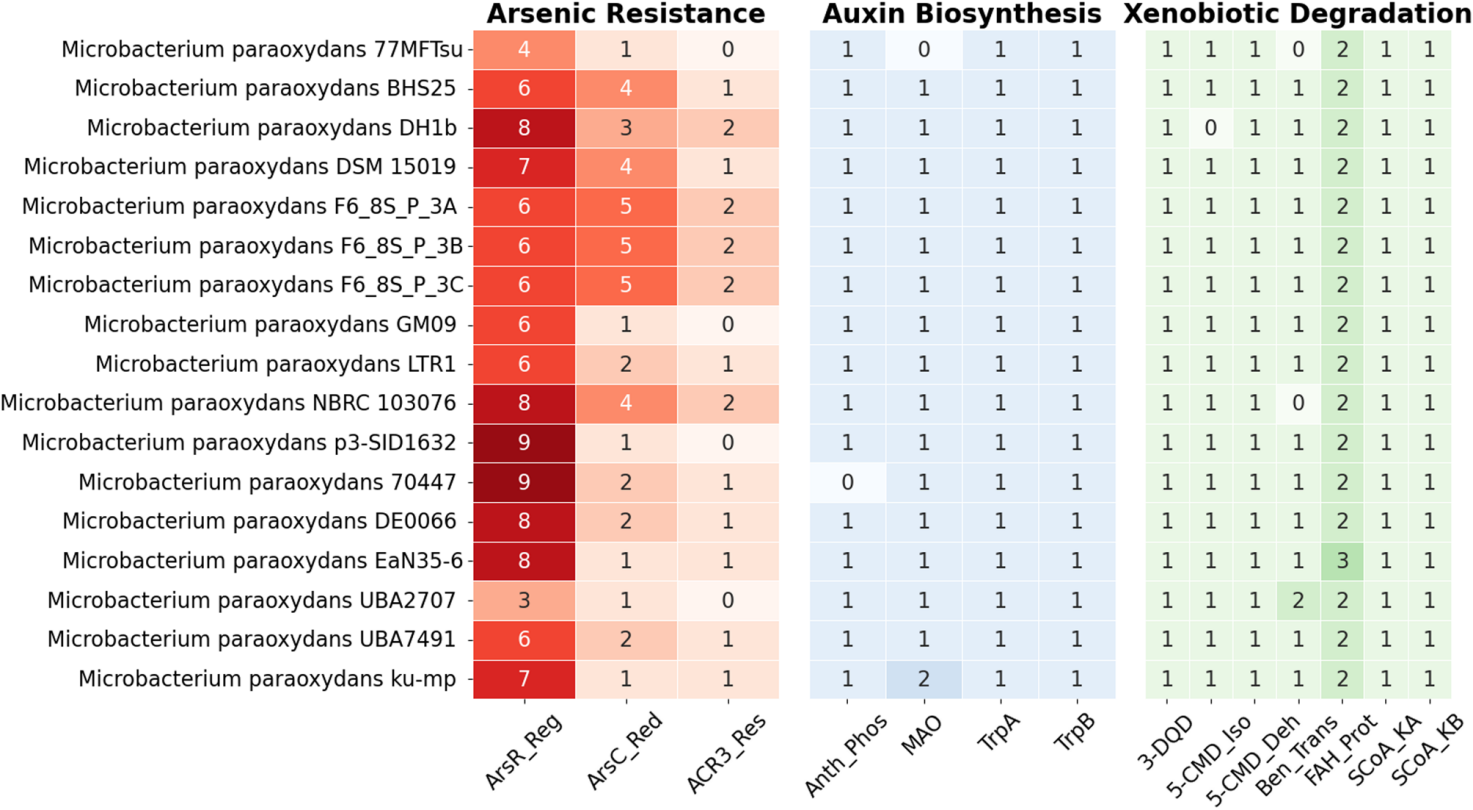
The number of genes involved in arsenic resistance, auxin biosynthesis and xenobiotic degradation that is present in the 17 *microbacterium* isolates

The three genes involved in arsenic resistance differed in number among the *M. paraoxydans* (Fig. 8). *M. paraoxydans* BHS25 possesses 6 *ars*R genes involved in the regulation of arsenic resistance, 4 *ars*C genes involved in arsenic reduction and 1 arsenic resistance gene, *acr*3. The genome closely related to *M. paraoxydans* BHS25 (LTR1 and F6_8S isolates) presented almost the same number of arsenic resistance regulatory genes, whereas the numbers of other genes seemed to differ. The number of arsenic resistance-related genes in the distant isolates (*M. paraoxydans* ku-mp, *M. paraoxydans* EaN35-6 and *M. paraoxydans* 77MFTsu3.2) fluctuated. In the case of auxin biosynthesis and xenobiotic degradation, the number of genes remained almost the same in all of the genomes of *Microbacterium paraoxydans.*

**Fig. 9:**
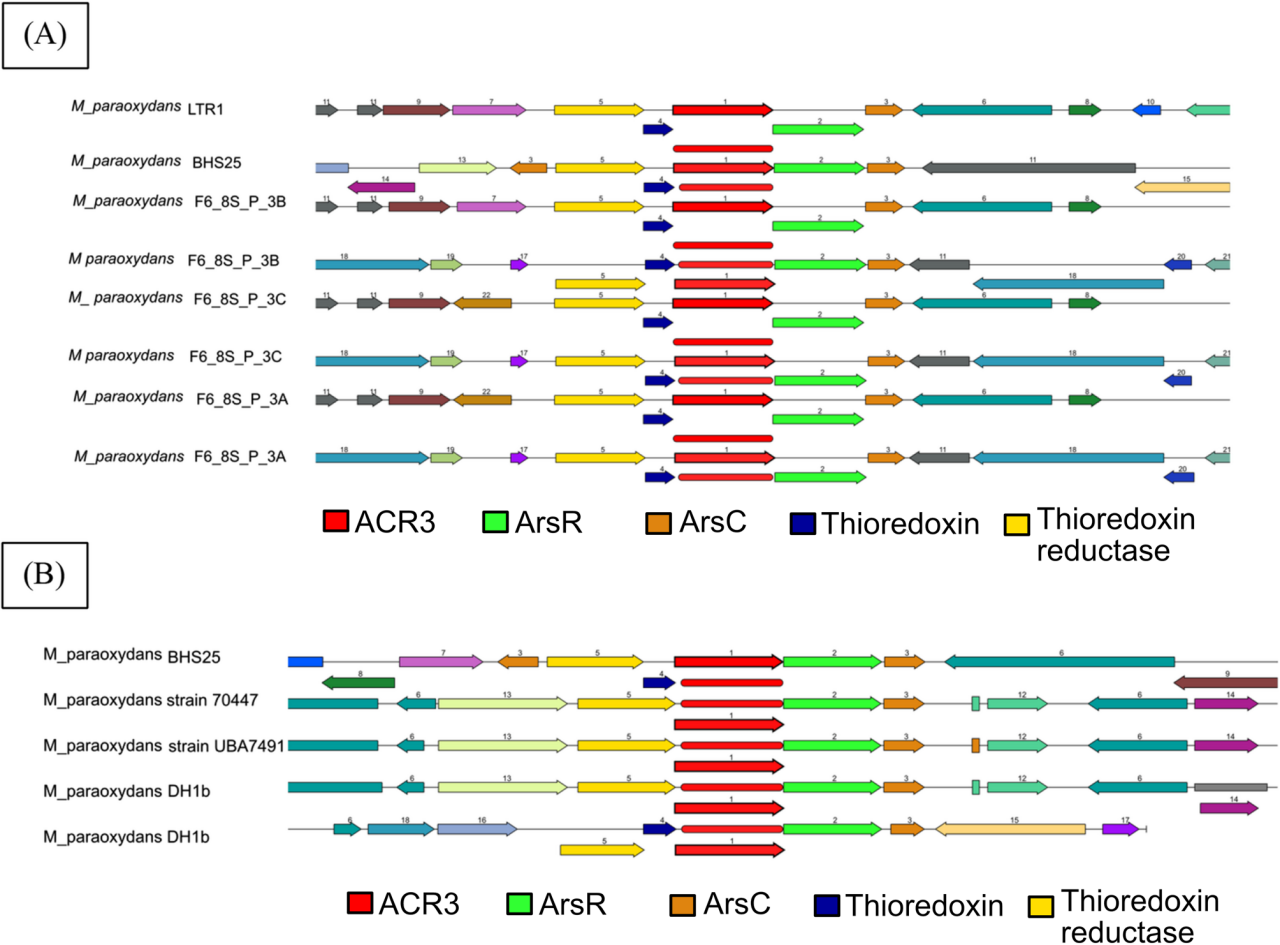
Synteny view of the arsenic resistance operon of (**A**) phylogenetically related and (**B**) phylogenetically distant isolates

Synteny analysis revealed that the arsenic resistance operon was conserved in both closely related and distant genomes, maintaining the same orientation across different species (Fig. 9A, B). This conservation suggested that the genetic architecture of arsenic detoxification was fundamental and likely driven by selective pressures to adapt to arsenic-contaminated environments. However, there was notable variation in the number of operons among different genomes. For example, the *M. paraoxydans* isolate F6_8S possessed two arsenic resistance operons, which could have resulted from gene duplication events or horizontal gene transfer. In contrast, *M. paraoxydans* 77MFTsu3.2 lacked an arsenic resistance operon.

## Discussion

### *Microbacterium paraoxydans* BHS25 is resistant to multiple environmental stresses

The genome of *Microbacterium paraoxydans* BHS25 revealed a diverse array of heavy metal resistance genes, highlighting its potential for bioremediation in arsenic-contaminated environments such as those found in Bangladesh. The presence of multiple arsenic resistance genes (*arsC_1*,* arsC_2*,* arsC_3*,* arsB*,* and acr3*) was particularly noteworthy, as these genes encode proteins involved in the reduction of arsenate to arsenite and the subsequent efflux of arsenite out of the cell, thereby conferring resistance to arsenic toxicity. Bacteria harbouring the *arsC* gene can reduce arsenate to arsenite. Although arsenite is more toxic, its greater mobility facilitates easier uptake by other bacteria [30]. These bacteria can metabolize arsenite or further detoxify it through processes such as methylation, converting it into less harmful forms [31]. The claim was supported by the fact that the BHS25 isolate was retrieved from an extremely arsenic-contaminated site in Bogura, Bangladesh and it showed arsenic transformation potential during initial culture screening of potential arsenotrophic bacteria.

Additionally, the detection of genes related to cadmium, zinc, and cobalt resistance (*czcD_1*,* czcD_2*,* czcR*) suggested that *M. paraoxydans* BHS25 can tolerate and possibly detoxify environments contaminated with these heavy metals [31, 32]. This finding was further supported by the presence of copper resistance genes (*copB_1*,* csoR*,* cutC*,* and ctpG*), which indicated the ability of bacteria to manage copper stress [33, 34]. The detection of phosphate regulatory genes (*phoB*,* pstA*,* pstC*,* pitA_1*,* pitA_2*) suggested a proficient phosphate uptake and utilization system, which is crucial in nutrient-limited conditions often observed in contaminated environments [35, 36]. The presence of the *vanY* gene within the *vanB* cluster, associated with vancomycin resistance, raises important considerations regarding the potential spread of antibiotic resistance genes in environmental isolates [37].

### The isolate has metabolic diversity to address global pollution challenges

*Microbacterium paraoxydans* BHS25 demonstrated adaptability and bioremediation potential in contaminated environments, with genes supporting carbohydrate and amino acid metabolism, energy production, and heavy metal resistance. Its metabolic pathways, including secondary metabolite biosynthesis and nutrient transport, highlighted its versatility and environmental resilience [38].

The bacterium also showed genetic potential for producing bioactive compounds, including antibiotics such as streptomycin, novobiocin, and penicillin, although further validation is needed to confirm its active production. Additionally, its genes related to xenobiotic degradation pathways suggested the ability to detoxify toxic compounds, making it a promising candidate for sustainable environmental management.

### The isolate has biotechnological potential to produce auxin

The presence of genes such as *trp*A and *trp*B, encoding the α and β subunits of tryptophan synthase, respectively, and *trp*D, encoding anthranilate phosphoribosyltransferase, indicated the bacterium’s capacity to synthesize tryptophan, a precursor for auxin (indole-3-acetic acid, IAA) production. Additionally, the presence of the *mao* gene, encoding monoamine oxidase, suggested potential involvement in auxin biosynthesis, as monoamine oxidase can participate in the oxidative deamination of tryptamine, leading to IAA production. These results correspond with the study [7], where the genomes having these genes were experimentally validated.

### Comparative genome analysis with globally reported *Microbacterium paraoxydans*

Comparative genomic analysis of the genomes of *Microbacterium paraoxydans* BHS25 and 17 other *M. paraoxydans* highlighted their evolutionary relationships and genetic diversity. *M. paraoxydans* BHS25 was closely related to isolates such as *M. paraoxydans* LTR1 and the three F6_8S strains, suggesting a shared origin and similar genetic traits. In a recent study, *M. paraoxydans* LTR1 was shown to be closely related to a novel bacterial species isolated from the International Space Station, which can survive in extreme conditions [39]. This similarity suggested that *M. paraoxydans* BHS25 might also be resistant to many harsh conditions.

Phylogenetic analysis revealed significant genetic differences among *M. paraoxydans* isolates such as *M. paraoxydans* DH1b, *M. paraoxydans* 70,447, *M. paraoxydans* UBA7491, and *M. paraoxydans* UBA2707, which formed distinct clades, revealing their evolutionary divergence from the main lineage. This diversity likely reflects adaptations to different environments [40, 41].

Pangenome analysis supported the above findings by identifying a cluster of closely related isolates with similar gene profiles, whereas distant species displayed a more unique gene matrix [42]. These unique genes might be considered further evidence of the similarities and diversity based on genes. Although the genome data are insufficient to definitively classify the pangenome as open or closed, the matrix in this study aligned with an open pangenome [43]. This finding indicated substantial genetic diversity within the *M. paraoxydans* species.

### Comparison at the protein level

The *arsC*,* arsR*, and *acr*3 genes help bacteria survive in arsenic-contaminated environments. arsC converts toxic arsenate (As(V)) into arsenite (As(III)). *arsR* is a regulator that activates arsenic resistance genes only when arsenic is present. acr3 pumps arsenite out of the cell, reducing its toxicity. Together, these genes form a system that detoxifies and removes arsenic. And this is the very simple operon to be expressed in most arsenic-resistant bacteria [44, 45].

The genes trpB, trpA, Anth_phos, and MAO are involved in tryptophan biosynthesis, which is a key step in auxin (IAA) production. trpA and trpB work together in the final step of making tryptophan, while Anth_phos helps in an earlier step. MAO may affect tryptophan levels indirectly. These genes suggest the bacteria might produce auxin if other necessary pathways are also present. In [7] five genes essential for auxin biosynthesis are mentioned. BHS25 possesses all of them, indicating that this bacterium has a strong potential for auxin production.

Several enzymes, including FAH, 3-dehydroquinate dehydratase II, benzoate transport protein, and catechol 2,3-dioxygenase, are reported to help bacteria break down aromatic compounds [46–49]. These enzymes enable the use of complex molecules like benzoate, catechol, and mandelate as energy sources. This shows the bacteria can degrade various environmental pollutants, highlighting their ecological importance.

*M. paraoxydans* BHS25 shared a close genetic relationship with isolates such as the Russian LTR1 and U.S. F6_8S strains, indicating that these isolates share similar arsenic resistance capabilities. BHS25, for example, carried six *ars*R genes for arsenic regulation, along with four *ars*C genes and one *acr*3 gene for arsenic reduction and efflux, highlighting its potential for arsenic detoxification [50].

Overall, the number of arsenic resistance genes varies among the genomes, likely reflecting adaptation to different environmental arsenic levels [51] In contrast, auxin synthesis genes are almost uniformly present, suggesting a conserved functional role. However, these genes do not appear in the core genome due to strict core criteria, which require presence in all genomes [11].

Synteny analysis revealed that the arsenic resistance operon is conserved across both closely related and distant genomes, emphasizing its critical role in adapting to arsenic stress [52]. However, the number of operons varied: F6_8S strains possessed two operons, likely due to gene duplication or transfer [52, 53]. These variations highlighted the evolutionary dynamics of *M. paraoxydans*, driven by inheritance and gene acquisition.

## Conclusion

This study presents the first reported whole genome of *Microbacterium paraoxydans* from Bangladesh. The presence of various resistance and metabolic genes in *Microbacterium paraoxydans* BHS25 indicates its ability to survive in harsh environments and a high potential to produce valuable compounds. The complete genome information provides a strong basis to focus on this bacterium for the possibilities of many biotechnical and environmental inventions.

## Supplementary Information

Supplementary Material 1.

Supplementary Material 2.

## Data Availability

The genome sequence generated during this study has been deposited in the NCBI GenBank under GenBank accession number JBBBVW000000000. The datasets used and/or analyzed during the current study are available from the corresponding author upon reasonable request.

## Abbreviations

ANI: Average Nucleotide Identity
BacMet: Antibacterial Biocide- and Metal-Resistance Genes Database
BLASTp: Basic Local Alignment Search Tool for proteins
BV-BRC: Bacterial and Viral Bioinformatics Resource Center (formerly PATRIC)
CARD: Comprehensive Antibiotic Resistance Database
CDS: Coding Sequences
COG: Clusters of Orthologous Groups
DSM: Deutsche Sammlung von Mikroorganismen (German Collection of Microorganisms)
FAH: Fumarylacetoacetate Hydrolase
GC: Guanine-Cytosine (content)
IAA: Indole-3-Acetic Acid
KEGG: Kyoto Encyclopedia of Genes and Genomes
L50: Contig length metric (half of the genome is in contigs of this size or larger)
N50: Assembly quality metric (median contig length)
NCBI: National Center for Biotechnology Information
Prokka: Prokaryotic Genome Annotation Tool
RAST: Rapid Annotation using Subsystem Technology
ResFinder: Tool for identifying antimicrobial resistance genes
SPAdes: St. Petersburg Genome Assembler
VFDB: Virulence Factor Database
WGS: Whole-Genome Sequencing
3-DQH: 3-Dehydroquinate Dehydratase
MAO: Monoamine Oxidase
Anth. Phos: Anthranilate Phosphoribosyltransferase
FAH: Fumarylacetoacetate Hydrolase
